# Artificial Triterpenoid Fatty Acid Ester Isolated From the Leaves of *Phytolacca icosandra* L

**DOI:** 10.1007/s13659-020-00249-x

**Published:** 2020-06-05

**Authors:** Elier Galarraga, Andersson Mavares, Neudo Urdaneta, Rafael E. Rodríguez-Lugo, Juan Manuel Amaro-Luis

**Affiliations:** 1 Departamento de Química. Edificio de Química Y Procesos, Universidad Simón Bolívar (USB), Apartado 89000. Caracas-1080A, Venezuela, USA; 2 Laboratorio de Química Bioinorgánica, Centro de Química, Instituto Venezolano de Investigaciones Científicas (IVIC), Venezuela, Caracas 1020-A USA; 3 Laboratorio de Productos Naturales, Departamento de Química. Facultad de Ciencias, Universidad de Los Andes (ULA), Mérida, Venezuela-5101, USA

**Keywords:** *Phytolacca icosandra*. triterpenoid. fatty acid ester. NMR. artificial products. BSLA

## Abstract

**Abstract:**

The methanol extract form the leaves of *Phytolacca icosandra* L., afforded the unprecedented artificial triterpenoid fatty acid ester **1** derived from the new natural triterpenoid phytolaccagenic acid 3-*O*-myristate (**1a**), along with the three known triterpenoids serjanic, acinosolic and phytolaccagenic acid (**2** – **4**). Their structures were stablished by HR-EI-MS, 1D and 2D NMR techniques. The possible mechanistic formation of **1** is proposed, and the in vitro toxicity of all compounds was assessed using the brine shrimp lethality assay (BSLA).

**Graphic Abstract:**

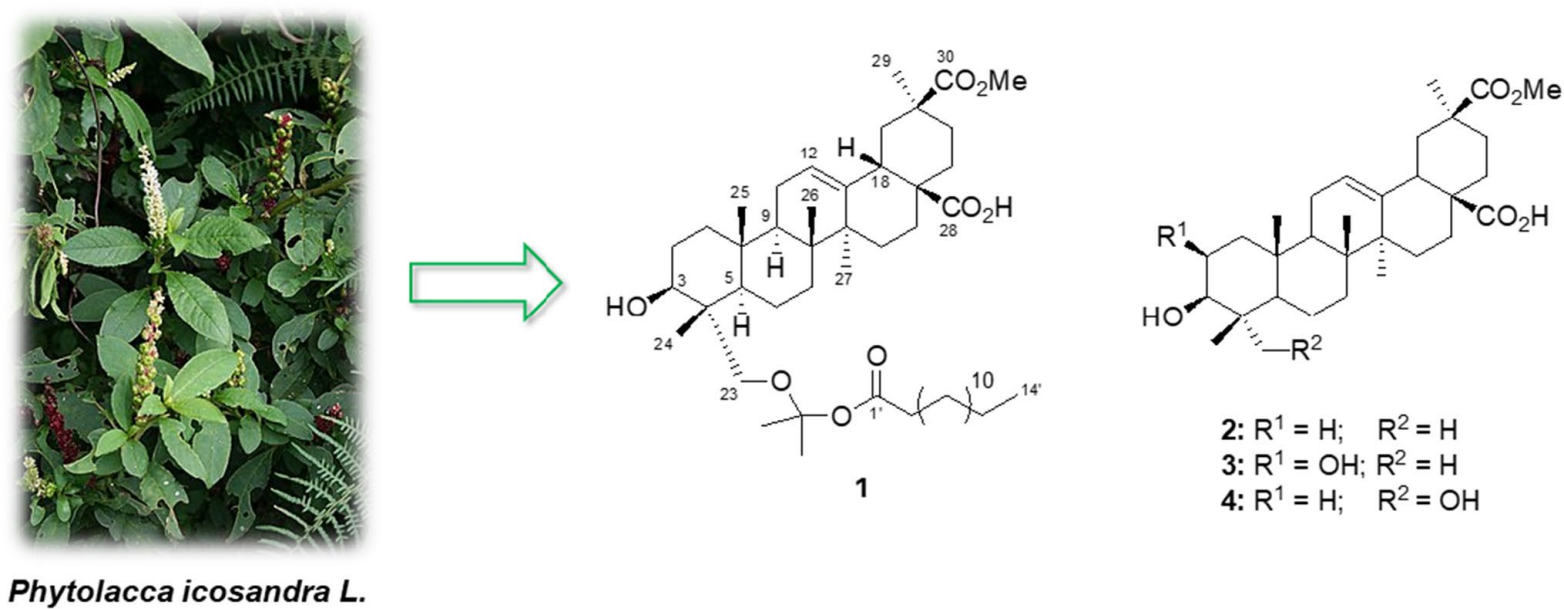

**Electronic supplementary material:**

The online version of this article (10.1007/s13659-020-00249-x) contains supplementary material, which is available to authorized users.

## Introduction

The chemistry of *Phytolacca ssp.* is fairly wide and comprises a variety of secondary metabolites, composed mainly by triterpenoids, flavonoids and lignans [1]. Plants belonging to genus *Phytolacca* have been used in folk medicine for the treatment of several affections such as edema, rheumatism and dermatitis [1–3]; also as a molluscicidal plant in schistosomiasis prevention and control [4, 5]. Several studies on *P. icosandra* have reported its antisecretory, anthelmintic, ovicidal and larvicidal activity [6–8].

**Fig. 1:**
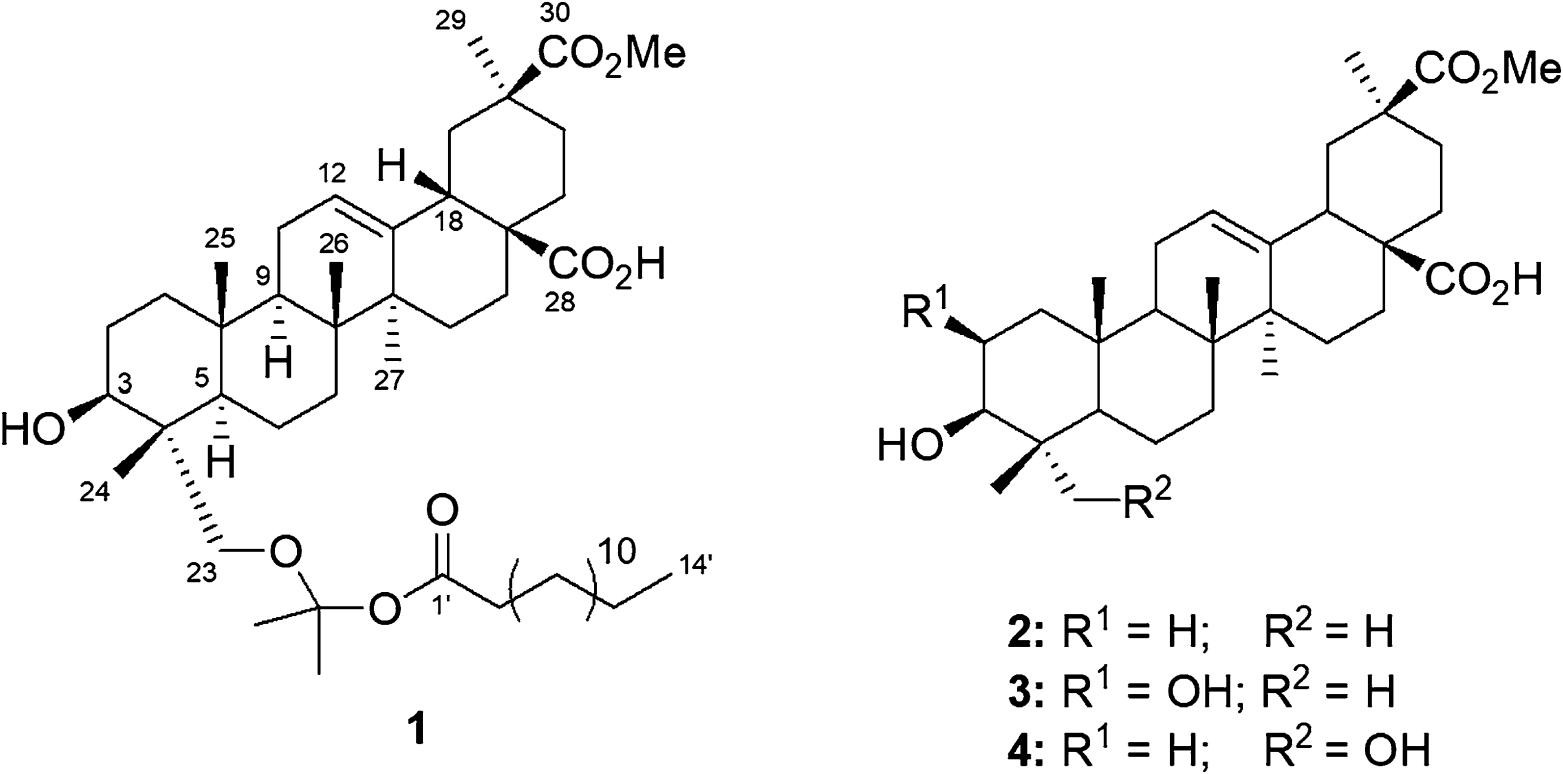
Chemical structure of compounds **1**—**4**

Phytochemical analysis of *P. icosandra* has lead to the isolation of several serjanic and spergulagenic acids [9, 10] and a previous investigation of the fruits yielded a novel peltogynoid, together with triterpenoids, neo-lignanes and 6′palmitoyl-*α*-*d*-glucoside sterols [11]. As part of our continuing search for new bioactive constituents from plants of the *Phytolacca* genus, the methanolic extract of the leaves of *P. icosandra* was investigated. As a result, a new artificial triterpenoid fatty acid ester (**1**) was isolated along with three other known pentacyclic triterpenoids **2**–**4** (Fig. 1). We also comment on the possible formation of **1**, and the in vitro toxicity of all compounds against brine shrimps (*Artemia salina*).

## Results and Discussion

### Structure Elucidation of Isolated Compounds

Compound **1** was isolated as a white wax. A molecular formula of C_48_H_80_O_8_ was assigned from its HR-MS spectra, which showed a molecuar ion peak [M]^+^ at *m/z* 784.5859 (calcd. 784.5853) accounting for nine degrees of unsaturation. The IR spectrum exhibited absorption bands due to the presence of hydroxyl and carboxylic acid groups (2900–3400 cm^−1^), carbonyl groups (1701–1705 cm^−1^), olefinic bond (1472 cm^−1^) and long chain alkanes band (728 cm^−1^). The ^1^H NMR spectrum in conjunction with the HMQC spectrum, revealed the presence of five tertiary methyl groups at *δ*_H/C_ 0.69/16.8 (H-26/C-26), 0.93/16.5 (H-25/C-25), 1.01/12.4 (H-24/C-24), 1.12/25.9 (H-27/C-27), 1.13/28.3 (H-29/C-29); a 2,2-dioxy-propane group [*δ*_H/C_ 1.39/29.7 (H-3′'/C-3′') and 1.42/19.3 (H-2′'/C-2′') and *δ*_C_ 99.0 (O > C < O, C-1′'), one methoxy group at *δ*_H_ 3.67 / *δ*_C_ 51.8, one oxymethine proton at *δ*_H_ 3.48 (1H, dd, *J* = 3.7, 11.7 Hz, H-3)/*δ*_C_ 77.6 (C-3), two oxymethylene protons at *δ*_H_ 3.41, 3.50 (2H, d, *J* = 10.7 Hz, H-23)/*δ*_C_ 72.6 (C-23) and one vinylic proton at *δ*_H_ 5.32 (1H, t, *J* = 3.5 Hz, H-12)/*δ*_C_ 123.2. Additional data showed that compound **1** was esterified with a long chain fatty acid, due the presence of several peaks between *δ*_H_ 1.20 – 1.32 (14H, m, H-5′ – H-11′)/*δ*_C_ 29.2 – 29.7, (C-5′ – C-11′), two multiplets at *δ*_H_ 1.22 (H-4′)/*δ*_C_ 29.0 (C-4′) and *δ*_H_ 1.59 (H-3′)/*δ*_C_ 24.7 (C-3′), one methylene triplet at *δ*_H_ 2.31 (2H, t, *J* = 7.4 Hz, H-2′)/*δ*_C_ 34.0 (C-2′) and one primary methyl at *δ*_H_ 0.85 (3H, t, *J* = 6.9 Hz, H-14′)/*δ*_C_ 14.4 (C-14′).

The ^13^C NMR spectra showed that compound **1** has an 3,23-dihydroxy-olean-12(13)-en-28,30-dioic acid-30-methyl ester triterpene skeleton, because in addition to the presence of the peaks assigned to the five tertiary methyl mentioned above, it is possible to locate peaks corresponding to six sp^3^ quaternary carbons [*δ*_C_ 36.8 (C-4), 37.2 (C-10), 39.4 (C-8), 41.4 (C-14), 43.7 (C-20), 45.8 (C-17)], three sp^3^ methines [*δ*_C_ 42.2 (C-18), 47.7 (C-9), 51.5 (C-5)], one oxymethylene carbon [*δ*_C_ 72.6 (C-23)], one oximethyne [*δ*_C_ 77.6 (C-3)], two carbons from a tri-substituted double bond [*δ*_C_ 123.2 (C-12), 142.8 (C-13)], and two carbonyl carbons [*δ*_C_ 176 (C-30), 183.4 (C-28) ppm]. An acyclic acetonide moiety, previously described as a 2,2-dioxy-propane group, esterified by the aforementionated long chain fatty acid were also elucidated in the molecule, across the ^13^C NMR spectra.

The HMBC correlations of the oxymethylene protons at *δ*_H_ 3.41/3.50 with carbons at *δ*_C_ 12.4 (C-24), 36.8 (C-4), 51.5 (C-5) and 77.6 (C-3), permitted to assign this protons to H-23. The acetonide moiety was located on C-23 due to the ^3^* J* HMBC interaction of H-23 protons with the carbon at *δ*_C_ 99.0 (C-1") and the interaction of this carbon with two tertiary methyl protons at *δ*_H_ 1.39 (H-3") and 1.42 (H-2"). Proton H-3 (*δ*_H_ 3.48) was assigned by its HMBC correlations with carbon peaks at *δ*_C_ 12.4 (C-24), 23.2 (C-2) and 72.6 (C-23). Surprisingly, there was no HMBC correlation between proton H-3 and the carbonyl group of the fatty acid at *δ*_C_ 179.7 (C-1′). The fact that the chemical shift of this proton at *δ*_H_ 3.48 was unusually shielded in comparison to acylated oleanane triterpenes at H-3 position, which are observed between *δ*_H_ 4.46–4.57 [12–16], indicated that the ester moiety of the fatty acid was not located at C-3 position. In view of these observations, the only available position for the fatty acid chain previously stated, would be at the isopropylienedioxy carbon (C-1") attached to C-23. Thus, the acetonide triterpenoid fatty acid ester **1** was elucidated as 3*β*,23*α*-dihydroxy-olean-12(13)-en-28,30-dioic acid-30-methyl ester-23,1"-isopropyl enedioxy-1"-tetradecanoate. Complete stereochemistry of the triterpene was confirmed by analysis of its NOESY spectrum along with some biogenetic and chemotaxonomic considerations. NOE interactions where detected between H-3/H-5/H-23 and H-24/H-25/H-26, interaction between H-18/H-30 was not observed thus confirming configuration at C-3 3*β*OH, junctions of the B/C rings "*trans*" (8*β*Me, 9*α*H) and the D/E rings "*cis*" (18*β*H; 28*β*COOH). This is also congruent with the configuration of all triterpenes previously isolated from *Phytolacca* genus.

**Fig. 2:**
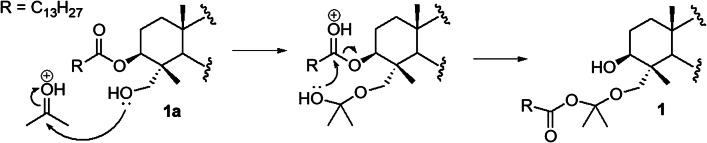
Possible formation mechanism for compound **1**

Although few, there has been some reports on natural occurring triterpenoidal acetonides from plants [17–19]. Despite the fact that **1** is considered unusual being an acyclic acetonide ketal, it is assumed to be an artifact derived from phytolaccagenic acid 3*β*-*O*-myristate (**1a**) during the chromatographic process, in which acetone was used as solvent [20, 21]. The proposed mechanism in the formation of **1** from **1a** involves firstly a nucleophilic attack of the C-23 hydroxyl to a protonated acetone molecule, followed by an intramolecular nucleophilic substitution at the fatty acid carbonyl at C-3 (Fig. 2).

Finally, the toxicity of all compounds was assayed in the brine shrimp lethality assay [22] and compound **1** exhibited mild toxicity against *Artemia*; results are shown in Table 1.

**Table 1:** Toxicity of compounds **1**–**4** to brine shrimps

	Sample			
	1	2	3	4
LC_50_ (µM)	33.5	85.3	22.1	15.1

## Experimental Section

### General Procedures

Optical rotation was measured in Karl-ZEISS, Model 93,772 equipment. IR spectra were obtained from KBr pellets with Shimadzu IR-408 equipment. Solution ^1^H, ^13^C{^1^H}, ^1^H,^1^H-COSY, HSQC, HMBC and NOESY NMR spectra were recorded on Bruker Avance 600 MHz spectrometer at *Laboratorio Nacional de Resonancia Magnética Nuclear*, *Instituto Venezolano de Investigaciones Cientificas* (IVIC), using CDCl_3_ and MeOD as solvent. Peak positions are relative to tetramethylsilane for ^1^H and ^13^C{^1^H}. The chemical shifts (*δ*) were measured according to IUPAC [23], expressed in parts per million (ppm) and were calibrated against the residual solvent resonance (^1^H) or the deuterated solvent triplet (^13^C). Coupling constants *J* are given in Hertz (Hz) as absolute values. The multiplicity of the signals are indicated as s, d, t, q, or m for singlets, doublets, triplets, quartets or multiplets respectively. All NMR spectra were recorded at room temperature (25 °C) in CDCl_3_ dried over molecular sieves. ESI–MS was run on a TSQ QUANTUM, Ultra AM, ThermoScientific Spectro-photometer and the HR-EI-MS analysis was conducted in a JEOL JMS-AX505WA spectrometer with direct inlet and dual approach mass analyzer, using electron impact (EI) method. Solvents were obtained from Sigma-Aldrich (Milwaukee, Wisconsin, USA) and Merck (Kenilworth, NJ, USA), and were used without any purification. Analytical thin layer chromatography (TLC) was performed on silica gel (15–40 µm PF_254_) 0.25 mm and 0.5 mm thick plates respectively (supplied by Merck), and the spots were visualized by spraying with AcOH/H_2_O/H_2_SO_4_ (37:8:5) mixture, followed by heating to 100 °C. Column chromatography was performed using silica gel 230–400 Mesh.

### Plant Material

*Phytolacca icosandra* leaves were collected in Mucuhies-Gavidia, Municipio Rangel, Estado Mérida-Venezuela, in August 2008 and identified by Ing. For. Juan Carmona Arzola, Universidad de Los Andes (Mérida-Venezuela). A *voucher specimen* (Amaro et al. N° 2322) was deposited in the MERF herbarium, Faculty of Pharmacy, ULA.

### Extraction and Isolation

Air-dried and powdered leaves of *P. icosandra* (≅ 2 kg) were exhaustively extracted at room temperature with MeOH in a Soxhlet for 48 h. After vacuum evaporation of the solvent, the crude extract (≅ 300 g) was pre-absorbed on normal phase silica gel and submitted to a chromatographic process (CC), using Hex/CHCl_3_ (0% up to 100%), Hex/EtOAc (30% up to 100%) and CHCl_3_/MeOH (20% up to 100%) mixture solvents, to afford 13 sub-fractions (A-M). Sub-fraction “E” (12.7 g, Hex/CHCl_3_ 80%) was submitted to further CC, using solvent mixture CHCl_3_/Acetone (19:1 *v*/*v*) as eluent, to afford six sub-fractions E_1_–E_6_. Compound **1** was isolated from sub-fraction E_2_ (1.16 g) trough chromatographic column process on silica gel and eluted with CHCl_3_/Acetone (9:1 *v*/*v*) solvent mixture, to yield a white wax (35.2 mg). A portion of fraction “I” (1.23 g, Hex/EtOAc 75%), was further fractioned and purified by several chromatographic processes (CC) on silica gel to afford **2** (36.7 mg) from sub-fraction I_2_ (CHCl_3_/Acetone, 17:3 *v*/*v*), **3** (28.7 mg) from sub-fraction I_3_(CHCl_3_/Acetone, 4:1 *v*/*v*), and **4** (60.2 mg) from sub-fraction I_5_ (CHCl_3_/Acetone 3:1 *v*/*v*).

### Identification of Known Compounds

Known compounds were identified by comparison of their physical constants and NMR spectroscopic data with those reported in the literature [24–26].

### ***Phytolaccagenic Acid 23α-O-Isopropyl Tetradecanoate*** (**1**)

White wax; [α]_D_^23^ + 23.8 (*c* 0.13, CHCl_3_); R_f_: 0.24 (CHCl_3_/Acetone, 9:1);. IR (KBr): 3500–2600, 2917, 1703–1705, 1472, 1206, 728 cm^−1^; ^1^H NMR (600 MHz, CDCl_3_) *δ* (ppm) 0.69 (3H, s, H-26), 0.75 (1H, m, H-5), 0.85 (3H, t, *J* = 6.9 Hz, H-14′), 0.93 (3H, s, H-25), 1.01 (3H, s, H-24), 1.12 (3H, s, H-27), 1.13 (3H, s, H-29), 1.20 – 1.39 (20H, m, H-4′/H-13′), 1.39 (3H, s, -CH_3_), 1.42 (3H, s, -CH_3_), 1.56 (1H, m, H-9), 1.59 (2H, m, H-3′), 2.31 (2H, t, *J* = 7.4, Hz, H-2′), 2.65 (1H, dd, *J* = 13.9, 13.7 Hz, H-18), 3.41 (1H, d, *J* = 10.7 Hz, H-23a), 3.48 (1H, t, *J* = 3.7, 11.7 Hz, H-3), 3.50 (1H, d, *J* = 10.7 Hz, H-23b), 3.65 (3H, s, -OCH_3_), 5.32 (1H, t, *J* = 3.5 Hz, H-12). ^13^C NMR (150 MHz, CDCl_3_) *δ* (ppm) 12.4 (CH_3_, C-24), 14.4 (CH_3_, C-14′), 16.5 (CH_3_, C-25), 16.8 (CH_3_, C-26), 17.6 (CH_2_, C-6), 19.3 (CH_3_, C-2′'), 22.7 (CH_2_, C-11), 23.0 (CH_2_, C-16), 23.2 (CH_2_, C-2), 23.4 (CH_2_, C-13′), 24.7 (CH_2_, C-3′), 25.9 (CH_3_, C-27), 27.5 (CH_2_, C-15), 28.3 (CH_3_, C-29), 29.0 (CH_2_, C-4′), 29.2 – 29.7 (CH_2_, C-5′/C-11′), 29.7 (CH_3_, C-3′'), 30.3 (CH_2_, C-21), 31.9 (CH_2_, C-12′), 32.7 (CH_2_, C-7), 33.4 (CH_2_, C-22), 34.0 (CH_2_, C-2′), 36.4 (C, C-4), 37.2 (C, C-10), 38.8 (CH_2_, C-1), 39.4 (C, C-8), 41.4 (C, C-14), 42.0 (CH_2_, C-19), 42.2 (C, C-18), 43.7 (C, C-20), 45.8 (C, C-17), 47.7 (CH, C-9), 51.5 (CH, C-5), 51.8 (-OCH_3_), 72.6 (CH_2_, C-23), 77.6 (CH, C-3), 99.0 (O > C < O, C-1′'), 123.2 (CH, C-12), 142.8 (C, C-13), 176.9 (C, C-30), 179.7 (C, C-1′), 183.4 (C, C-28). HR-MS *m/z* 784.5859 [M^+^] (calcd for C_48_H_80_O_8_, 784.5853).

### Brine Shrimp Lethality Assay

The assay was performed as described previously by Meyer et al. [22] with some minor modifications. Brine shrimp eggs (Gulf Breeze®) were hatched in artificial sea water prepared with commercial salt mixture (Instant Ocean®), illuminated and oxygenated with an aquarium pump. After 48 h incubation at 27 °C, 10 shrimps were transferred with a Pasteur pipette to three sample vials for each of three doses (100, 50, 10 μg/mL) for a total of nine vials. The sample was prepared by dissolving the compound **1** (3 mg) in CHCl_3_ (5 mL) and transferring the solution to each vial (833, 417 or 83 μL solution for 100, 50 or 10 ppm doses) followed by high vacuum for 1 h. After the solvent was evaporated, the compound was redissolved in 20 μL of Tween 80® and 5 mL of artificial sea water were added to achieve the correct concentration. Survivors were counted and the percent deaths at each dose and control were determined. Tween 80® at this concentration did not affect this bioassay. The LC_50_ and 95% confidence intervals were calculated from 24 h counts, using the Probit analysis method [27].

## Electronic supplementary material

Below is the link to the electronic supplementary material.

Supplementary file1 (DOCX 1180 kb)

## References

[1] Williams LAD, Rösner H, Conrad J, Möller W, Beifuss U, Chiba K, Nkurunziza JP, Kraus W (2002). Rec. Res. Devel. Phytochem..

[2] Jolliffe G (1982). British Homeopath J..

[3] Ravikiran G, Raju AB, Venugopal Y (2011). Int. J. Res. Pharm. Biomed. Sci..

[4] Lemma A (1970). Bull. World Health Org..

[5] Lambert JDH, Wolde-Johannas L, Makhubu L (1985). Bioscience.

[6] J.A. Santos-López, J.R. Villagómez-Ibarra, A. López-Ramirez, G. Montiel-Jarillo, M. Bautista-Ávila, J.A. Gayosso-de Lucio, C. Velázquez-González, Rev. CENIC Cienc. Biol. 41, 1–5 (2010)

[7] Hernández-Villegas MM, Borges-Argáez R, Rodriguez-Vivas RI, Torres-Acosta JFJ, Méndez-Gonzalez M, Cáceres-Farfan M (2011). Vet. Parasitol..

[8] Hernández-Villegas MM, Borges-Argáez R, Rodríguez-Vivas RI, Torres-Acosta JFJ, Méndez-González M, Cáceres-Farfán M (2012). Vet. Parasitol..

[9] Treyvaud V, Marston A, Dyatmiko W, Hostettmann K (2000). Phytochemistry.

[10] Galarraga E, Amaro-Luis JM, Rojas LB, Mitaine-Offer AC, Lacaille-Dubois MA (2014). Ciencia (Maracaibo).

[11] E. Galarraga Montes, J.M. Amaro-Luis, Nat. Prod. Res. 30, 89–94 (2016) 10.1080/14786419.2015.1038537 25942389

[12] Ukiya M, Akihisa T, Yasukawa K, Kasahara Y, Kimura Y, Koike K, Nikaido T, Takido M (2001). J. Agric. Food Chem..

[13] Wandji J, Tillequin F, Mulholland DA, Wansi JD, Fomum TZ, Fuendjiep V, Libot F, Tsabang N (2002). Planta Med..

[14] Shen YC, Prakash CVS, Wang LT, Chien CT, Hung MC (2003). J. Chin. Chem. Soc..

[15] Wang KW (2007). Nat. Prod. Res..

[16] Maza H, Mkounga P, Fenkama SL, Sado SK, Ishikawac H, Nishino H, Nkengfack EA (2017). Phytochem. Lett..

[17] F.J. Arriaga-Giner, J.M. Rullkötter, T.M. Peakman, E. Wollenweber, Z. Naturforsch. 46**c**, 507–512 (1991)

[18] Iskenderov DA, Isaev IM, Isaev MI (2009). Chem. Nat. Comp..

[19] Yang SX, Yu ZC, Lu QQ, Shi WQ, Laatsch H, Gao JM (2012). Phytochem. Lett..

[20] Maltese F, van der Kooy F, Verpoorte R (2009). Nat. Prod. Commun..

[21] Hanson JR (2017). J. Chem. Res..

[22] Meyer BN, Ferrigni NR, Putnam JE, Jacobsen LB, Nichols DE, McLaughlin JL (1982). Planta Med..

[23] Harris RK, Becker ED, Cabral de Menezes SM, Goodfellow R, Granger P (2001). Pure & Appl. Chem..

[24] Glombitza KW, Gielsdorf W, Eckhardt G, Koch ML (1975). Planta Med..

[25] W.S. Woo, Natural Products Research Institute, Seoul Natural University. (1978)

[26] Harkar S, Razdan TK, Waigth ES (1984). Phytochemistry.

[27] Finney D (1971). Probit Analysis, A Statistical Treatment of the Sigmoids Response Curve.

