# Supplementary material for: Artificial Triterpenoid Fatty Acid Ester Isolated From the Leaves of Phytolacca icosandra L
Source: Nat Prod Bioprospect. 2020 Jun 5;10(4):221–5. doi: 10.1007/s13659-020-00249-x (PMC7367978; doi:10.1007/s13659-020-00249-x)
Supplement: Supplementary file 1 — Supplementary file1 (DOCX 1180 kb) [file 13659_2020_249_MOESM1_ESM.docx]

**Supplementary Material**

**Artificial triterpenoid fatty acid ester isolated from the leaves of *Phytolacca icosandra* L.**

Elier Galarraga M.^a^*, Andersson Mavares^a^, Neudo Urdaneta^a^, Rafael Rodríguez and Juan Manuel Amaro-Luis^b^

^a^*Departamento de Química. Edificio de Química y Procesos. Universidad Simón Bolívar (USB). Apartado 89000. Caracas-1080A. Venezuela;* ^b^*Laboratorio de Productos Naturales. Departamento de Química*. *Facultad de Ciencias. Universidad de Los Andes (ULA). Mérida*, *Venezuela-5101*

(Tel.: +58 0212 9063983; e-mail: *eliergalarraga@usb.ve*)

**Abstract**

The methanol extract form the leaves of *Phytolacca icosandra* L., afforded the unprecedented artificial triterpenoid fatty acid ester **1** derived from the new natural triterpenoid phytolaccagenic acid 3-O-myristate (**1a**), along with the three known triterpenoids serjanic, acinosolic and phytolaccagenic acid (**2** – **4**). Their structures were stablished by HR-EI-MS, 1D and 2D NMR techniques. The possible formation of **1** is proposed and the *in vitro* toxicity of all compounds was assessed using the brine shrimp lethality assay (BSLA).

**Keywords:** *Phytolacca icosandra*, Triterpenoid fatty acid ester, NMR, Artificial products, BSLA.

**Table of Content**

**Figure S1:** HR-EI-MS (70 eV) spectrum of Compound **1**

**Figure S2:** ESI-MS (Positive ion mode) spectrum of Compound **1**

**Figure S2:** ^1^H-NMR spectrum of Compound **1**

**Figure S3:** ^13^C-NMR (BB) spectrum if Compound **1**

**Figure S4:** ^13^C-NMR-APT spectrum if Compound **1**

**Figure S5:** ^1^H-^1^H COSY spectrum of Compound **1**

**Figure S6:** HMQC spectrum of Compound **1**

**Figure S7:** HMBC spectrum of Compound **1**

**Figure S8:** Important HMBC correlations for compound **1**.

**Figure S9:** NOESY spectrum of Compound **1**

**Figure S10:** IR spectrum of Compound **1**

**Figure S11:** ^1^H NMR (600 MHz), ^13^C NMR (150 MHz) and HMBC data of **1**.

**Figure S1:** HR-EI-MS (70 eV) spectrum of Compound **1**


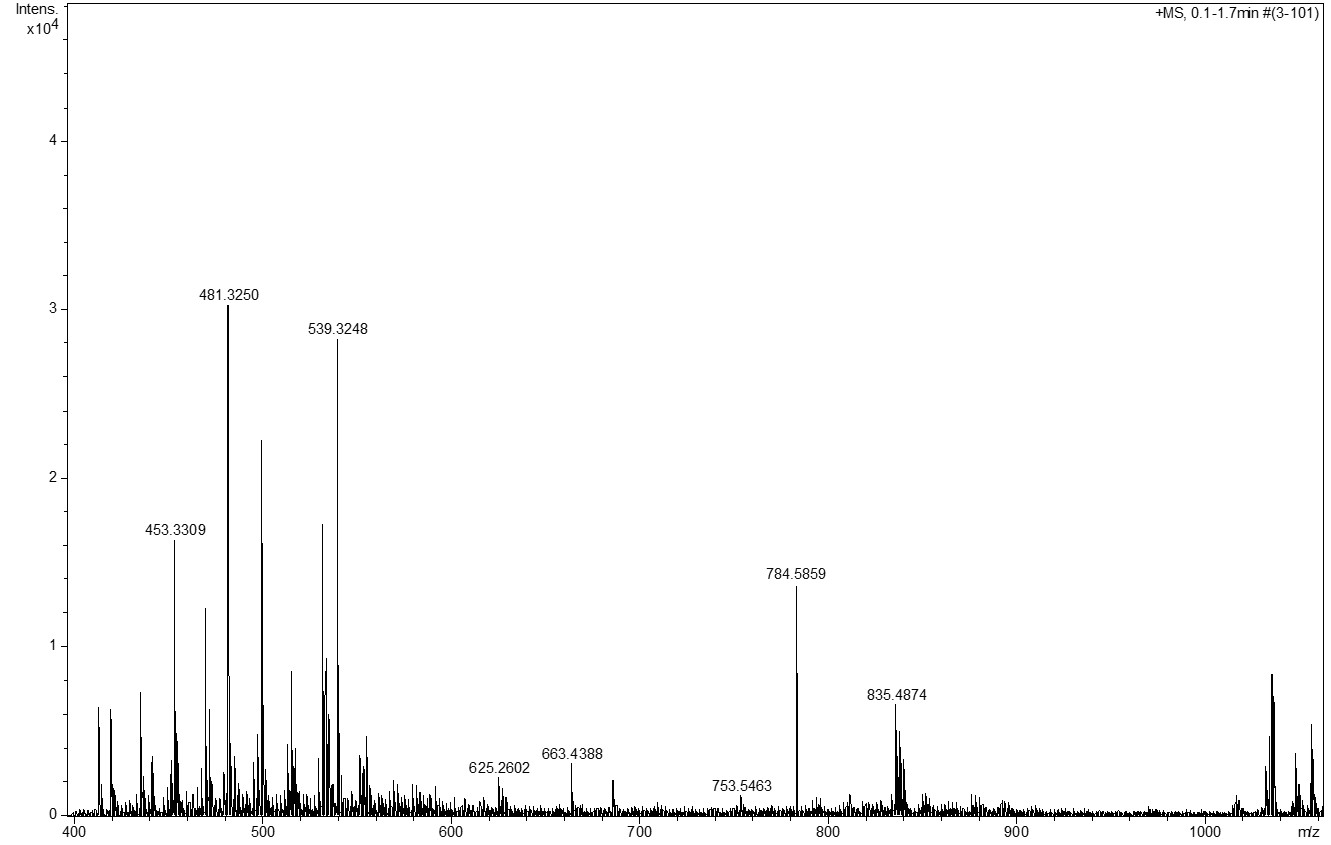


**Figure S2:** ESI-MS (Positive ion mode) spectrum of Compound **1**


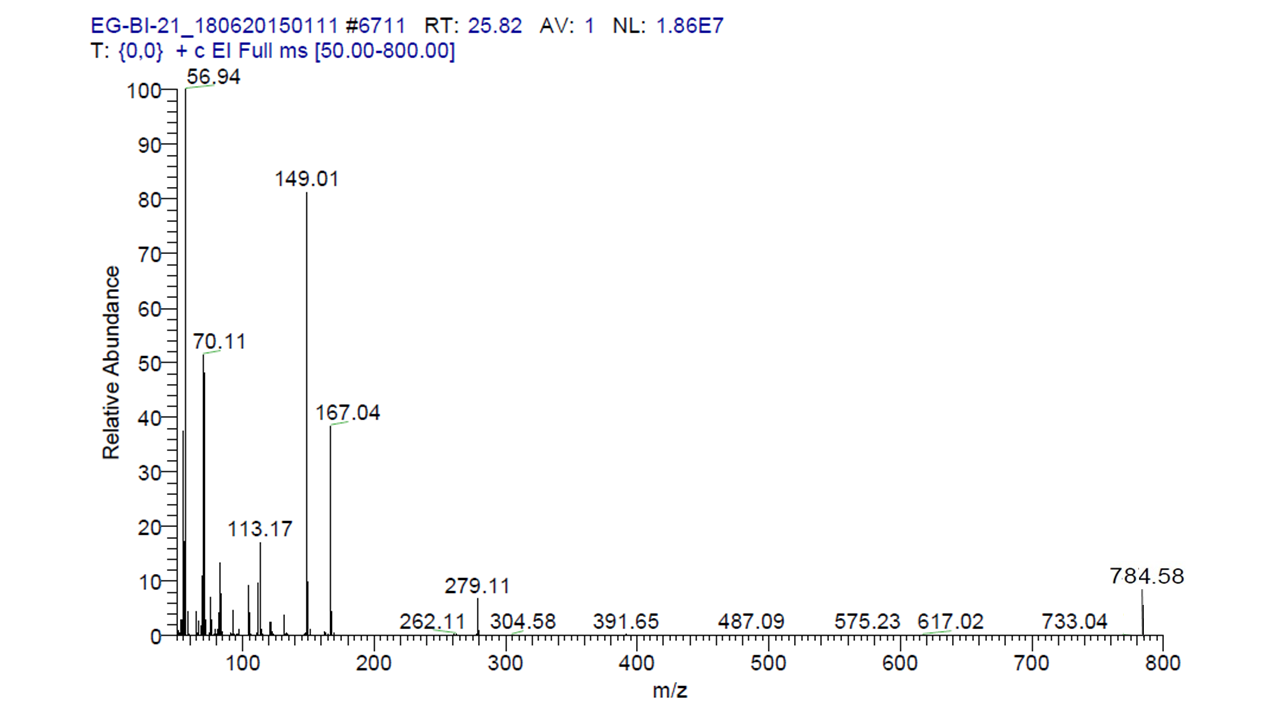


**Figure S2:** ^1^H-NMR spectrum of Compound **1**


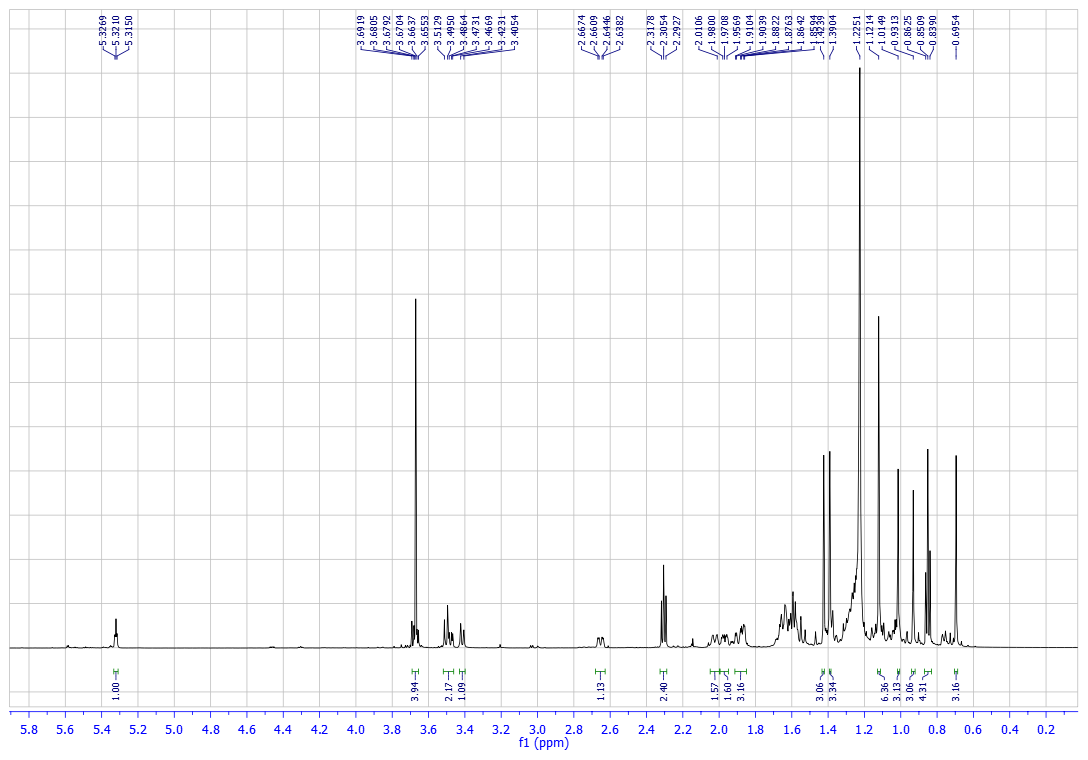


**Figure S3:** ^13^C-NMR (BB) spectrum of Compound **1**


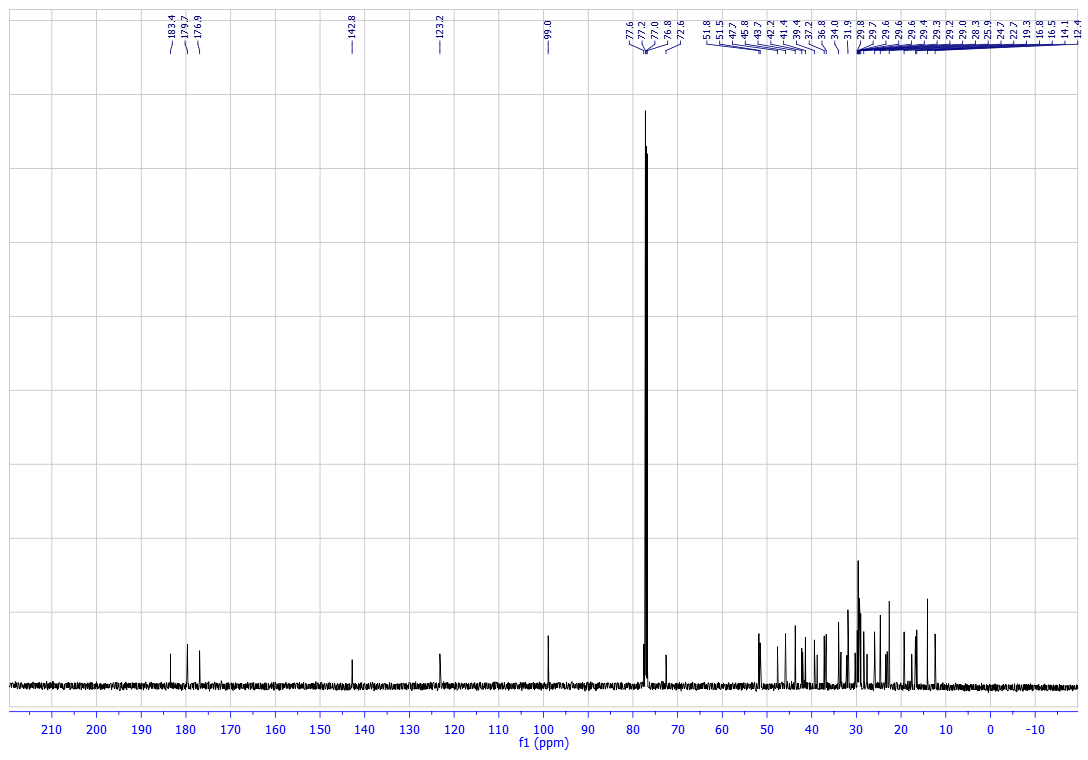


**Figure S4:** ^13^C-NMR-APT spectrum of Compound **1**


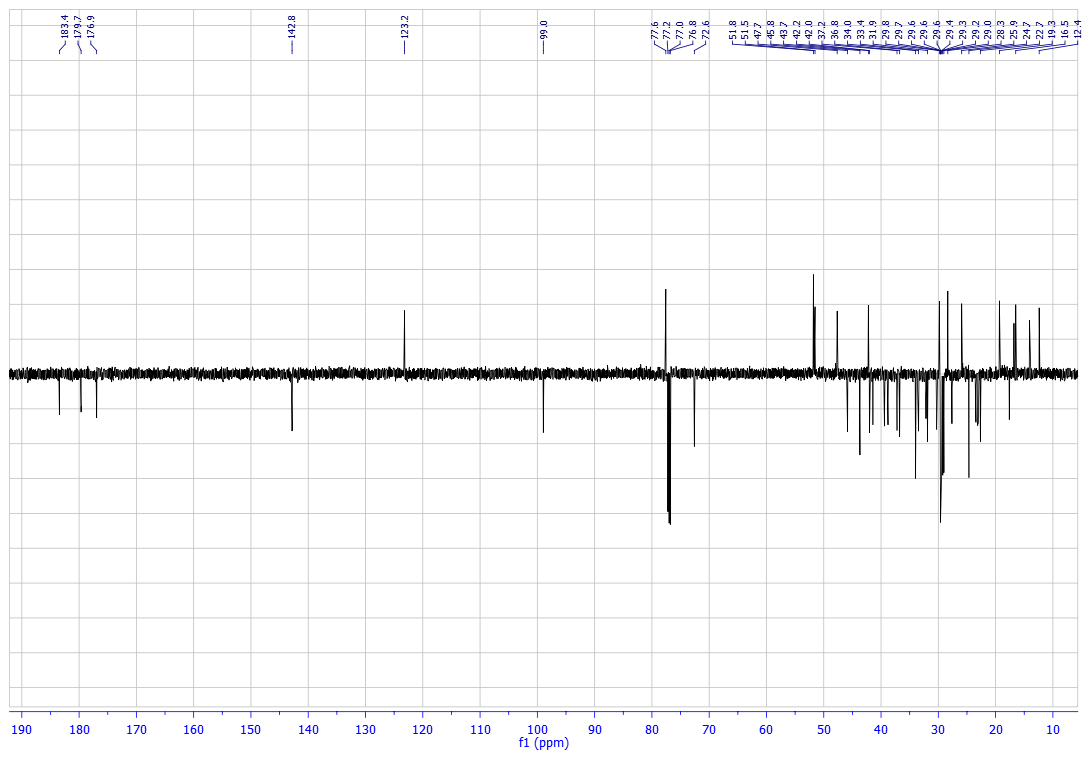


**
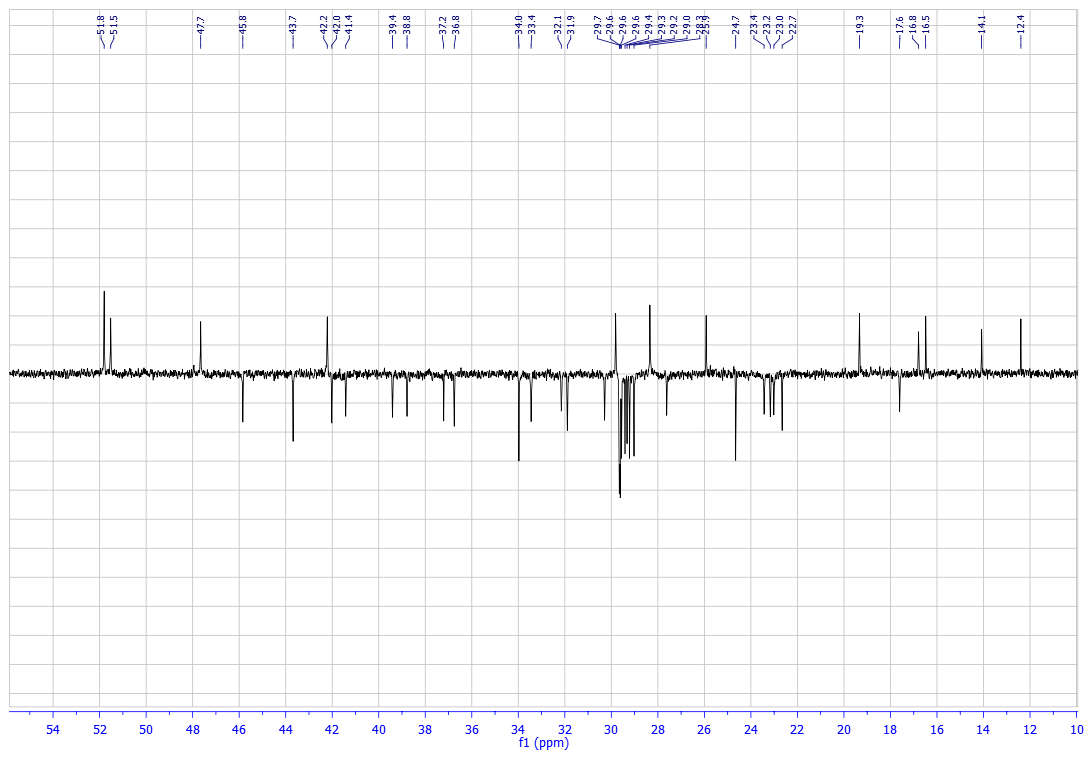
**

**Figure S5:** ^1^H-^1^H COSY spectrum of Compound **1**


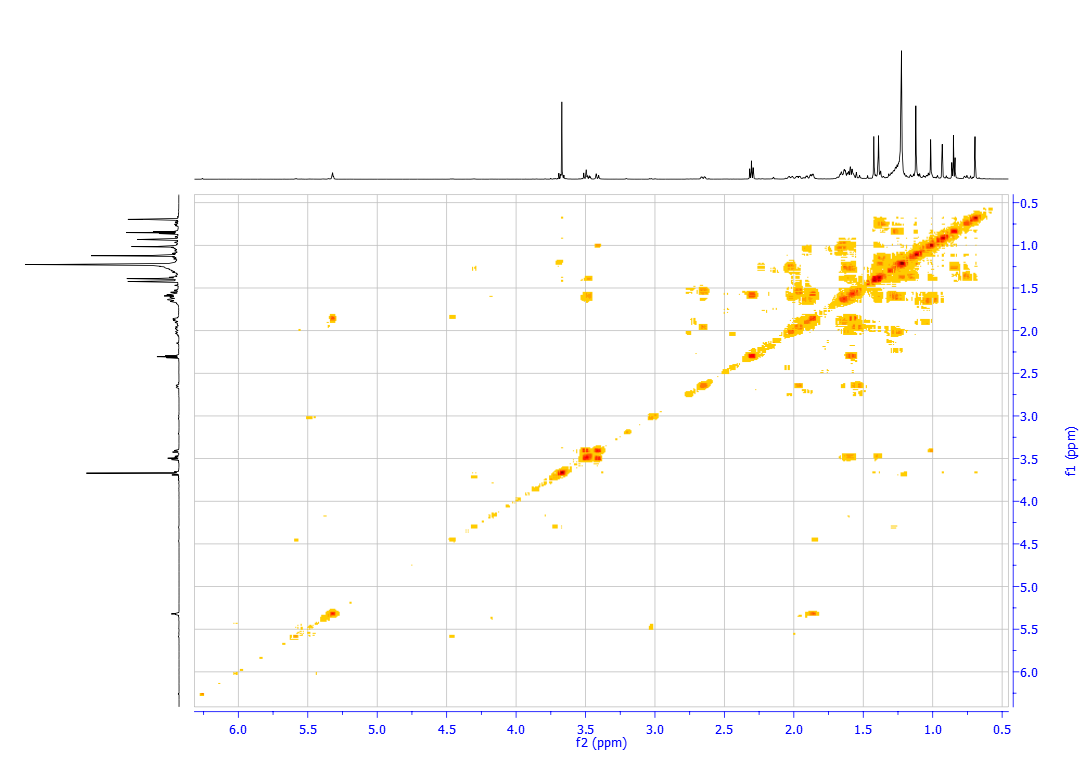


**Figure S6:** HMQC spectrum of Compound **1**


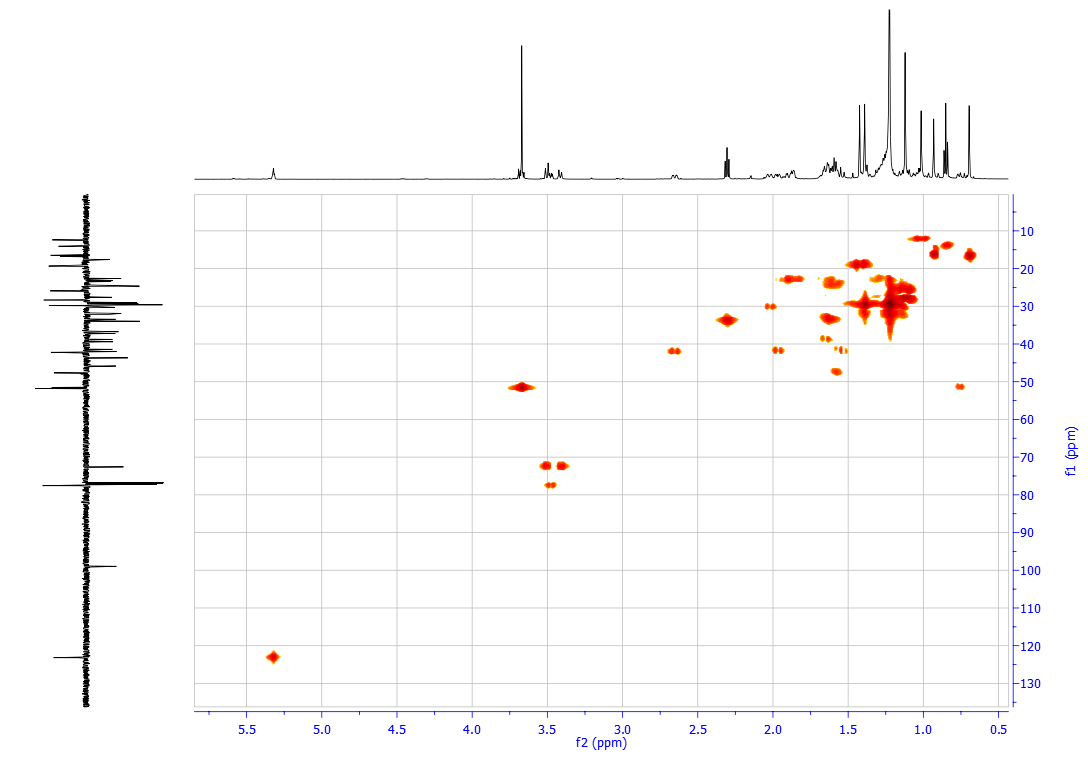


**Figure S7:** HMBC spectrum of Compound **1**


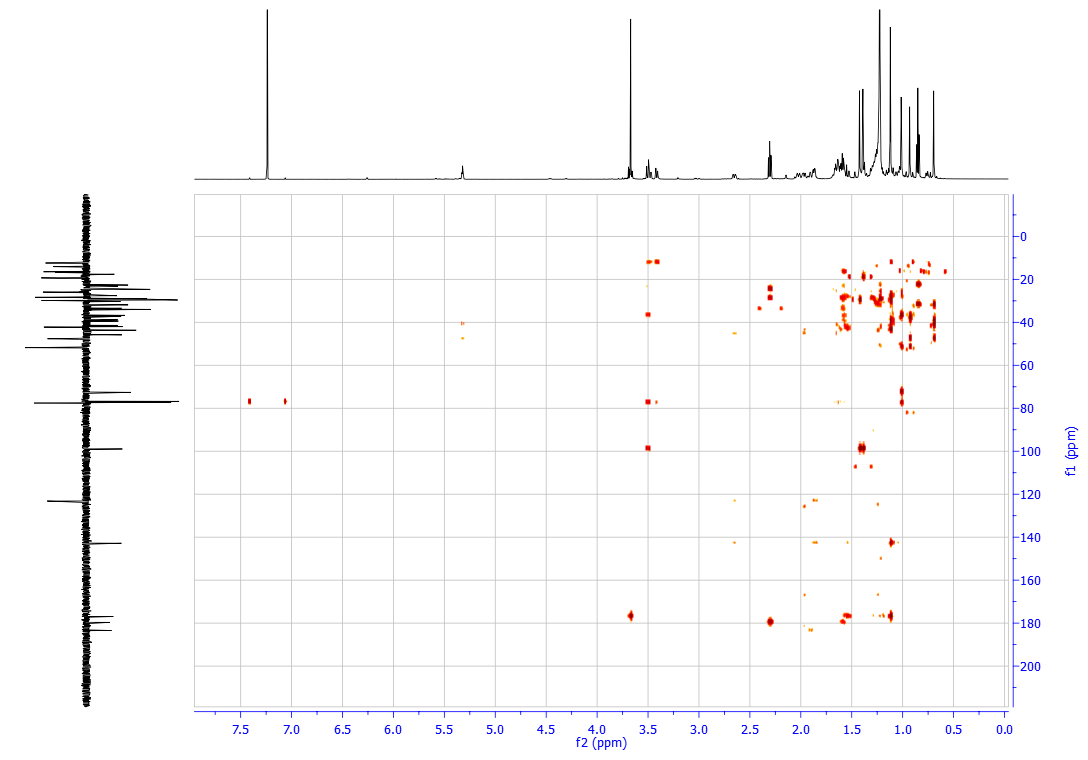


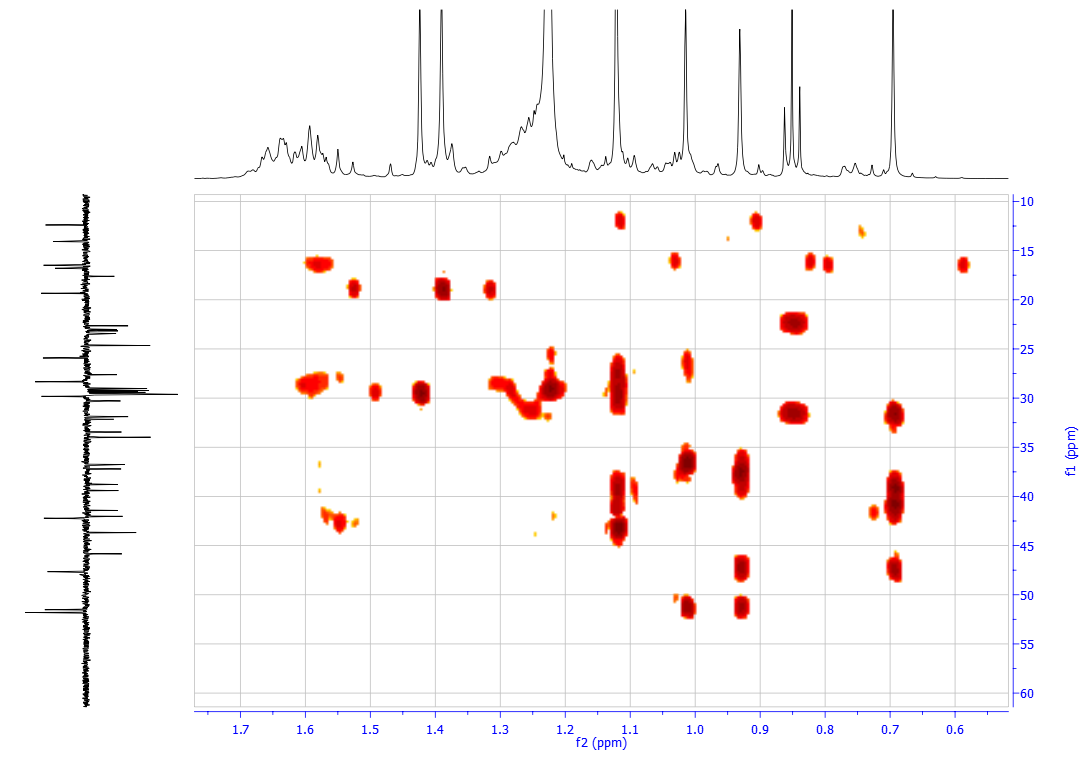


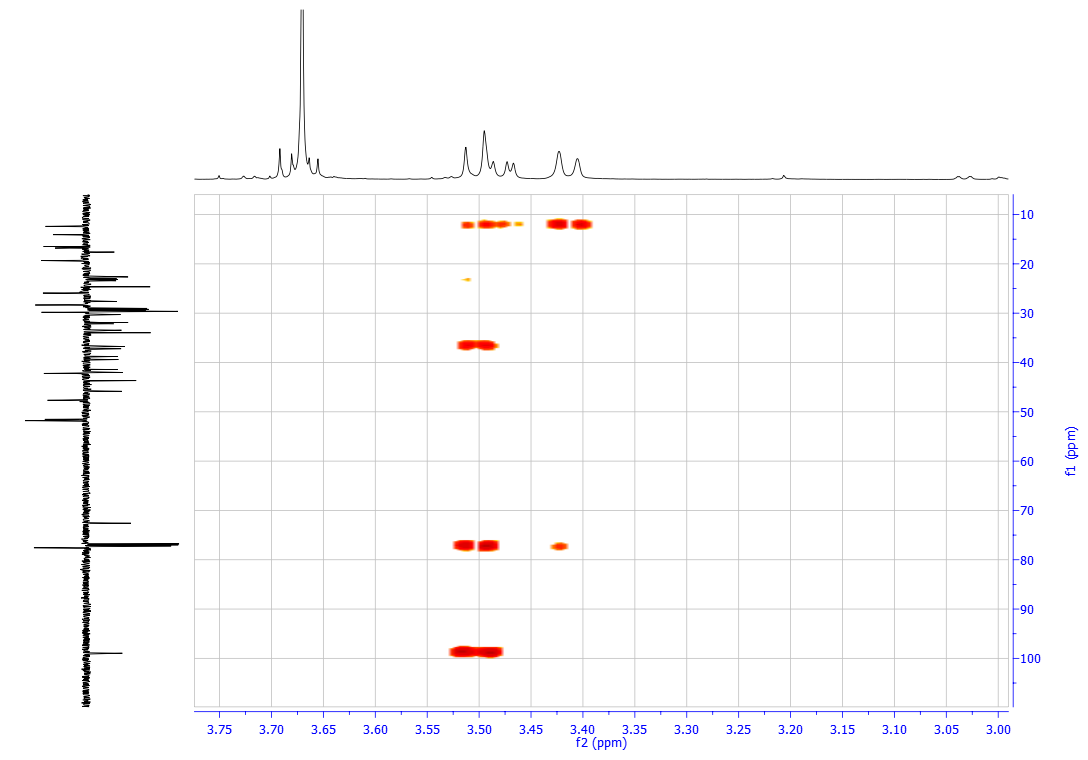


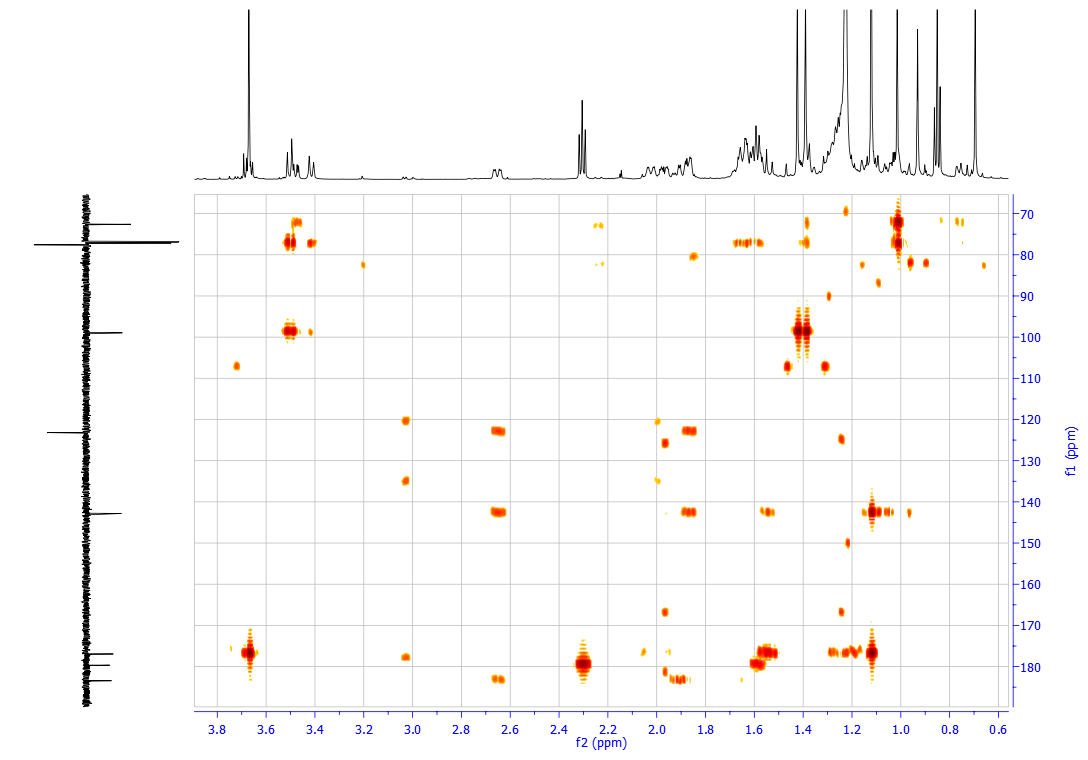


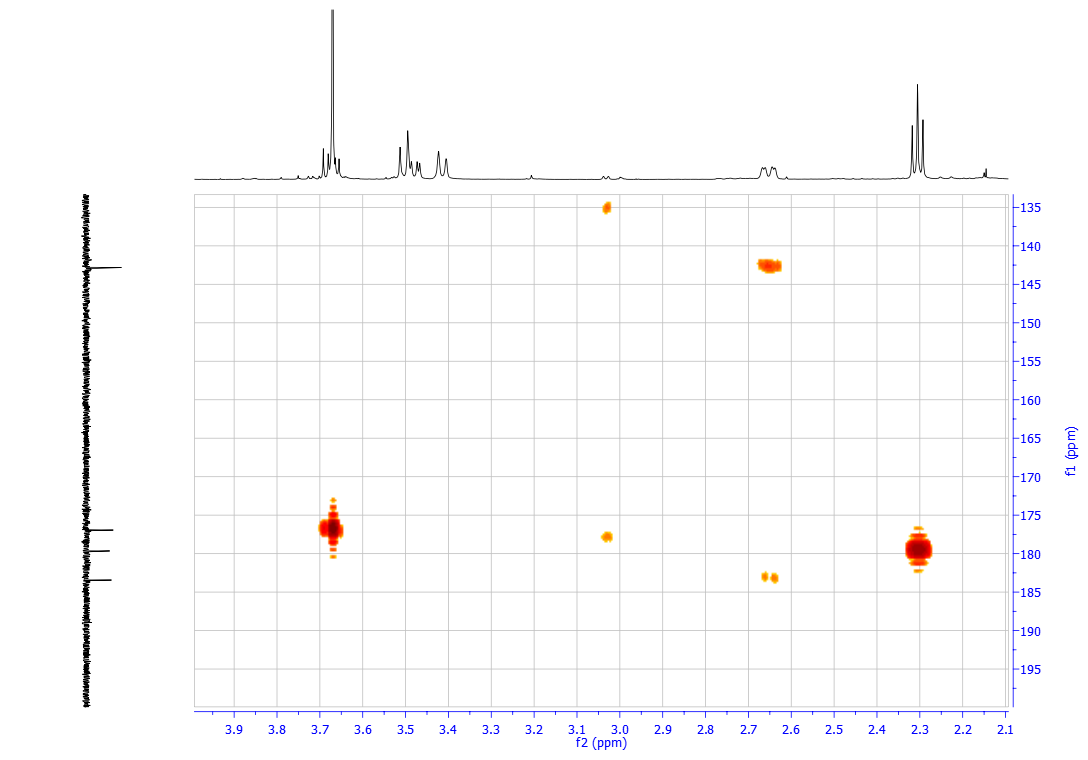


**Figure S8:** Important HMBC correlations for compound **1**.


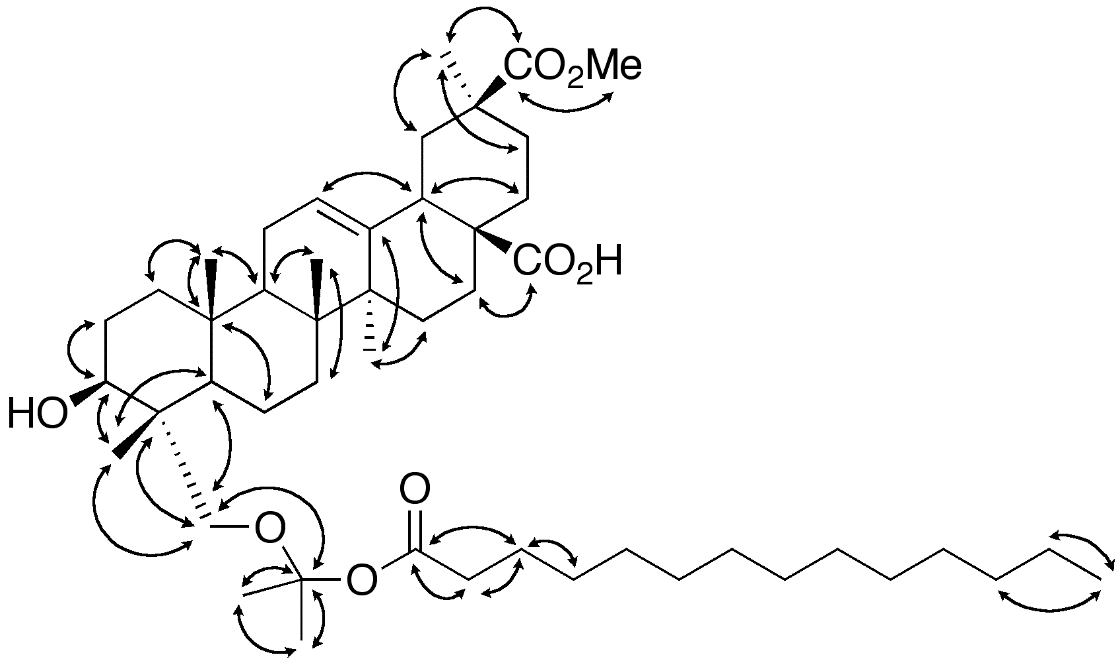


**Figure S9:** NOESY spectrum of Compound **1**

**Figure S10:** IR spectrum of Compound **1**

**Figure S11:** ^1^H NMR (600 MHz), ^13^C NMR (150 MHz) and HMBC data of **1**.

| N° | δ ^13^C^a^ | δ ^1^H (*mult,* *J* in Hz) | HMBC |
| --- | --- | --- | --- |
| 1 | 38.8 t | 1.65 (*m*), 1.22 (*m*) | C(3), C(25) |
| 2 | 23.2 t | 1.29 (*m*), 1.20 (*m*) | H(3) |
| 3 | 77.6 d | 3.48 (*t*, 3.7, 11.7) | C(2), C(23), C(24) |
| 4 | 36.8 s | - | H(23), H(24) |
| 5 | 51.5 d | 0.75 (*m*) | H(24) |
| 6 | 17.6 t | 0.69 (*m*), 0.92 (*m*) | H(5) |
| 7 | 32.1 t | 1.39 (*m*), 1.15 (*m*) | H(26) |
| 8 | 39.4 s | - | H(26), H(27) |
| 9 | 47.7 d | 1.56 (*m*) | H(12), H(25), H(26) |
| 10 | 37.2 s | - | H(25) |
| 11 | 22.7 t | 1.89 (*dd*, 3.9, 16) | H(12) |
| 12 | 123.2 d | 5.32 (*t*, 3.5) | C(9), C(11), C(14), H(18) |
| 13 | 142.8 s | - | H(18), H(27) |
| 14 | 41.4 s | - | H(12), H(26), H(27) |
| 15 | 27.5 t | 1.65 (*m*), 1.31 (*m*) | H(27) |
| 16 | 23.0 t | 1.87 (*m*), 1.62 (*m*) | H(18), C(28) |
| 17 | 45.8 s | - | H(18), H(19) |
| 18 | 42.2 d | 2.65 (*dd*, 3.9, 13.7) | C(12), C(16), C(17), C(22), C(28) |
| 19 | 42.0 t | 1.95 (*m*), 1.54 (*m*) | C(20), H(29), C(30) |
| 20 | 43.7 s | - | H(19), H(29) |
| 21 | 30.3 t | 2.02 (*m*), 1.15 (*m*) | H(29) |
| 22 | 33.4 t | 1.64 (*m*), 1.22 (*m*) | C(18) |
| 23 | 72.6 q | 3.41, 3.50 (*d*, 10.7) | C(3), C(4), C(5), C(24), C(1") |
| 24 | 12.4 q | 1.01 (*s*) | H(3), C(5), H(23) |
| 25 | 16.5 q | 0.93 (*s*) | C(1), C(5), C(9), C(10) |
| 26 | 16.8 q | 0.69 (*s*) | C(7), C(8), C(9), C(14) |
| 27 | 25.9 q | 1.12 (*s*) | C(8), C(13), C(14), C(15) |
| 28 | 183.4 s | - | H(16), H(18) |
| 29 | 28.3 q | 1.12 (*s*) | C(19), C(20), C(21), C(30) |
| 30 | 176.9 s | - | H(19), H(29), -OCH_3_ |
| -OCH_3_ | 51.8 q | 3.67 (*s*) | C(30) |
| 1''O>C<O | 99.0 s | - | H(23), H(2"), H(3") |
| 2'' | 19.3 q | 1.42 (*s*) | C(1"), C(3") |
| 3'' | 29.7 q | 1.39 (*s*) | C(1"), C(2") |
| 1' | 179.7 s | - | H(2'), H(3') |
| 2' | 34.0 t | 2.31 (*t*, 7.4) | C(1'), C(3'), C(4') |
| 3' | 24.7 t | 1.59 (*m*) | C(1'), H(2') |
| 4' | 29.0 t | 1.22 (*m*) | H(2'), H(5') |
| 5'-11' | 29.2 – 29.7 t | 1.20 – 1.32 (*m*) | - |
| 12' | 31.9 t | 1.39 (*m*), 1.22 (*m*) | H(14') |
| 13' | 23.4 t | 1.21 (*m*) | H(14') |
| 14' | 14.4 q | 0.85 (*t*, 6.9) | C(12'), C(13') |

^a^Multiplicity determined by APT and HMQC experiments (s = quaternary, d = methine, t = methylene, q = methyl)
